# Visual Antipriming Effect: Evidence from Chinese Character Identification

**DOI:** 10.3389/fpsyg.2017.01791

**Published:** 2017-10-17

**Authors:** Feng Zhang, Amanda J. Fairchild, Xiaoming Li

**Affiliations:** ^1^Department of Psychology, Henan University, Kaifeng, China; ^2^Department of Psychology, University of South Carolina, Columbia, SC, United States; ^3^Health Promotion, Education, and Behavior, University of South Carolina, Columbia, SC, United States

**Keywords:** antipriming, Chinese character identification, overlapping representation, visual cognition, priming

## Abstract

Marsolek et al. (2006) have differentiated antipriming effects from priming effects, by adopting a novel priming paradigm comprised of four phases that include a baseline measurement. The general concept of antipriming supports the overlapping representation theory of knowledge. This study extended examination of the Marsolek et al. (2006) paradigm by investigating antipriming and priming effects in a series of Chinese character identification tasks. Results showed that identification accuracy of old characters was significantly higher than baseline measurements (i.e., the priming effect), while identification accuracy of novel characters was significantly lower than baseline measurements (i.e., the antipriming effect). This study demonstrates for the first time the effect of visual antipriming in Chinese character identification. It further provides new evidence for the overlapping representation theory of knowledge, and supports generalizability of the phenomenon to Chinese characters.

## Introduction

Historically, there has been controversy in the literature as to whether knowledge representations are stored in a discrete or overlapping way. Recent research has increasingly provided support for the latter hypothesis of overlapping knowledge representation in the brain (Seidenberg and McClelland, 1989; Masson, 1995). Previous studies in the area have found that different objects (e.g., house, shoes, chairs) activate the same area of the human ventral temporal cortex (Ishai et al., 1999; Haxby et al., 2001), and that their representations undergo dynamic learning changes after initial usage (Sigala and Logothetis, 2002; Marsolek, 2003). This overlapping representation is helpful not only in improving knowledge storage efficiency, but also in recognizing new information (McClelland and Rumelhart, 1985).

Traditional theories of visual object recognition suggest that objects are recognized based on shape information (Bramão et al., 2011). For example, Biederman's (1987) recognition-by-components theory proposes that objects are represented in terms of geons, or basic geometric building blocks. Current evidence indicates that visual object shapes are stored within superimposed and modifiable representations in the brain. Most cells in the visual cortical area TE are sensitive to moderately complex features of whole objects, such that activation of different critical features is necessary to specify an object (Tanaka, 1993). In the ventral visual pathway, the representation of objects including occipital and temporal regions is a distributed representation of information about object form (Ishai et al., 2000). Moreover, the same neuron can respond to different classes of objects depending on their visual similarity (Riesenhuber and Poggio, 2002). The above studies support a model in which the representations of different classes of objects are distributed and overlapped.

Conventionally, repetition priming experimental procedures have been comprised of two phases: an initial encoding phase and a subsequent test phase (e.g., Cave and Squire, 1992; Lewis and Ellis, 1999). The research demonstrated that repeated stimuli were processed easily in the test phase, after initial coding. The theory of overlapping representations (Masson, 1995) would suggest that these results are more complex, however. Specifically, the examination of repetition priming effects using a two-phase experimental procedure such as that described above might conflate positive and negative results; exposure to priming stimuli in the encoding phase would not only facilitate subsequent processing of repetition-primed stimuli but would also impair subsequent processing of other stimuli sharing overlapping representations with repetition-primed stimuli (Marsolek, 2008). In line with this concern, Marsolek et al. (2006) proposed a novel, four-phase experimental paradigm including a baseline measurement to test repetition priming in a more complete framework. In the first phase of Marsolek et al.'s (2006) paradigm, participants heard the names of 50 objects and responded with like or dislike judgements based on their meanings. The purpose of this phase was to avoid visual processing of objects so that a pure baseline of visual identification could be achieved in the following phase. The second phase was an unbiased baseline test with no potential for repetition or antipriming, in which participants viewed and identified 100 objects that differed from those presented in Phase 1. In the third phase, participants viewed 50 objects (different from objects encountered previously) and made preference judgments on the stimuli. The last phase served as the object identification test. Here, participants viewed and identified 100 objects; half of them were the same objects presented in Phase 3 (i.e., primed objects), and the other half were novel objects that differed from any previously presented stimuli (i.e., antiprimed objects).

Studies demonstrating the antipriming effect lend further support for overlapping representation theory. Specifically, Marsolek et al. (2006) and Marsolek (2008) found that when objects (e.g., “piano”) were identified, their representations were enhanced such that it was easier to identify the same objects again; it was harder to identify novel stimuli (e.g., “desk”) that had overlapping representations with previous primes. This newly observed phenomenon was termed *antipriming*. Recent physiological research has found that as compared to primed objects, the ERPs elicited by antiprimed objects demonstrate a more positive waveform than baseline and primed objects in post-1,100 ms time windows, after the average behavioral RT of 855 ms (Marsolek et al., 2010). Marsolek et al. (2010) proposed that representations of antiprimed objects were weakened after processing similar primed objects, and that the late antipriming ERP effect reflected changes in representation of the antiprimed objects after initial processing. The latter was thought to reflect the ongoing adjustments of overlapping visual representations. Marsolek et al. (2006) and Marsolek (2008) argued that visual objects were stored in the form of overlapping presentation in the neo-cortex of the brain and that their representations changed dynamically.

Replication of the existing research in this area is necessary to better understand its effects, particularly as it relates to reproducibility of results demonstrated in different types of visual stimuli. Reproducibility, and the importance of replication studies, has received increasing attention in the last decade (e.g., Ioannidis, 2005, 2012; Pashler and Harris, 2012). Recently, findings from 100 studies previously published in several psychological journals (with statistically significant results) were replicated to examine reproducibility of findings. This work found that only 36% of the replication studies had significant results, calling the results of the original studies into question (Open Science Collaboration, 2015). With the aforementioned in mind, the present study aims to extend examination of the Marsolek et al. (2006) paradigm to investigate whether antipriming is a reliable effect, and to examine whether the effect is generalizable across different domains. Specifically, the current study considers the tenability of the antipriming effect in identification of Chinese characters.

Chinese characters are a combination of pronunciation, shape, and meaning. Chinese characters can be considered as verbal stimuli that are based on pictorial form, including different form components. Graphical information is the first lexical information accessed in Chinese word identification, and reading Chinese is a visual form-to-meaning process (Perfetti and Tan, 1998). Perceptual similarity has a significant impact on verbal stimuli in this context (Ly et al., 2013). Orthographic neighbors also have a significant effect on the identification of visually presented words (Andrews, 1997; Spataro et al., 2017). Given that traditional Chinese characters are a pictorial form language, it may be reasonable to infer that the visual antipriming effect in object identification as illustrated by Marsolek and colleagues can extend to Chinese character identification. The theory of overlapping representation would suggest that priming a Chinese character will cause difficulty in identifying another Chinese character that shares overlapping representations with the original prime. With this guiding hypothesis, we used Marsolek et al.'s (2006) experimental framework to evaluate whether the effect of visual antipriming holds in Chinese character identification.

## Method

### Participants

Twenty-two students (10 males, 12 females) at Henan University in China participated in the experiment in exchange for course credit. The average age of participants in the sample was 23.18 ± 2.41 years old. All individuals had normal (or corrected to normal) vision and normal hearing. Informed consent was obtained from each student prior to participation, and the experiment was performed in accordance with the ethical standards of the institutional research committee.

### Materials

Two hundred and fifty Chinese characters (frequency range: 1,507–3,373) were selected from the *Modern Chinese Character frequency List* (State Language Commission and China Standard Bureau, 1992). All chosen characters were commonly used and familiar to participants. There were no significant differences in strokes [*F*_(3, 246)_ = 0.377, *p* = 0.770] or frequency [*F*_(3, 246)_ = 0.001, *p* = 1.000] among the characters used in the four experimental phases (50 characters in Phase 1, 100 characters in Phase 2, 50 characters in Phase 3, and 100 characters in Phase 4: inclusive of 50 characters from Phase 3 and 50 new characters). The composition structures of the Chinese characters both in Phases 3 (the priming condition) and 4 (the test condition) included two parts, and one half of a primed character was the same as an antiprimed character, and the other half of the primed character was different from the antiprimed character. For example, a primed character was “枪” (gun). Accordingly the antiprimed character was “棉” (cotton). The two characters were made up of two parts in which the left part of the character was the same (“木”, meaning “wood”), and the right part of the character was different. For the primed and antiprimed characters, there were no semantic or phonological relations between them. In the antiprimed condition, the prime and the target were different characters and they were not of the same general category. A complete list of the Chinese characters used as experimental materials in the study is provided in the **Appendix**.

### Procedure

The experiment was conducted individually in a dimly lit, small room with a 17-inch display (vertical refresh rate: 75 Hz). Each participant completed a short practice trial to become familiar with the experimental procedure prior to conducting the formal experiment. The response keys were balanced among different participants. The viewing distance of the participants was approximately 60 cm. E-prime 2.0 software controlled the stimuli and recorded participant responses. The total experiment lasted approximately 30 min.

In line with the Marsolek et al. (2006) paradigm, four phases were included in the experiment. In the first phase, participants watched the fixation point “+” while listening to the pronunciations of 50 characters through headphones. For each character, they were asked to make preference judgments. If they liked the stimulus, they were asked to press the “1” key; if they did not like the stimulus, they were asked to press the “2” key. The fixation point and the characters both appeared in white against a blank background.

In the second phase, which was the baseline measurement, 100 characters were presented for 15 ms. Half of these characters were presented above the fixation point, and the other half were presented below the fixation point (the presentation locations were intermixed pseudo-randomly). All of the characters in this phase differed from those in the first phase. For each character, participants were told to identify it as correctly and quickly as possible and then to verbalize it aloud. At the same time, they were asked to press the space key so that response times could be recorded.

In the third phase, which was the visual encoding stage, 50 characters were presented that differed from those in the preceding phases. The characters appeared in the middle of the screen for 3,000 ms. This time period was chosen to allow for sufficient visual identification so that the visual form representations of these characters could be strengthened. For each character, participants were asked to make preference judgments as they did in the first phase. If they liked the stimulus, they were asked to press the “1” key; if they did not like the stimulus, they were asked to press the “2” key.

In the fourth phase, which was the testing stage, 100 characters were presented in the same manner as Phase 2 of the experiment. Half of the characters were identical to those used in the third phase (primed characters), and the other half of the characters were new characters (antiprimed characters). All characters in the phase were intermixed pseudo-randomly. For each character, participants were told to identify it as correctly and quickly as possible and then verbalize it aloud as they did in the second phase. At the same time, they were asked to press the space key so that response times could be recorded.

## Results

**Figure 1:**
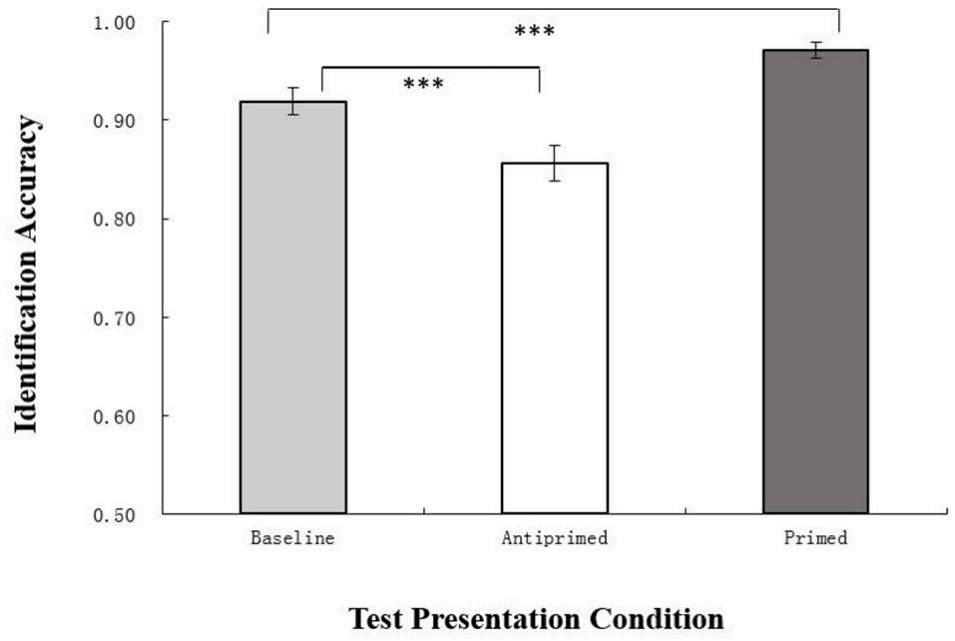
Mean character identification accuracy rates (±*SEM*) in baseline, antiprimed, and primed conditions are plotted as a function test presentation condition. The accuracy rates of antiprimed characters were significantly lower than baseline, and those of primed characters were significantly higher than baseline. ^***^p < 0.001.

Chinese characters identification accuracies in different experimental conditions are shown in Figure 1. Results of repeated-measures analysis of variance (ANOVA) showed that test presentation condition had a main effect [*F*_(2, 42)_ = 56.37, *p* < 0.001, η^2^_*p*_ = 0.73]. The results of *post-hoc* analyses using the least significant difference (LSD) criterion indicated that the identification accuracy of antiprimed characters (0.86) was significantly lower than baseline (0.92; *p* < 0.001); the identification accuracy of primed characters (0.97) was significantly higher than baseline (*p* < 0.001). These results indicate support for the visual antipriming effect in Chinese character identification.

Results from repeated-measures ANOVA on response times found no significant main effect of test presentation condition [*F*_(2, 42)_ = 1.14, *p* = 0.329]. The results of *post-hoc* analyses (LSD) indicated that both the antiprimed condition (557.39 ms) and the primed condition (511.23 ms) showed no significant difference from the baseline condition (513.56 ms) on response times (*p* > 0.05). However, the response times of the antiprimed condition were significantly higher than the primed condition (*p* = 0.002).

## Discussion

This is the first study to demonstrate a visual antipriming effect in Chinese character identification, providing additional evidence for the overlapping representation theory of knowledge. Traditionally, the human brain was thought to be a symbol processing system, such that representations of different items were stored in an independent or discrete way. The distributed representation theory, however, states that the representation of different items in the brain overlaps, and is supported by evidence for antipriming effects in empirical data. Following Marsolek et al.'s (2006) experimental paradigm, the present study explored the visual antipriming effect in Chinese character identification. Our study results aligned with previous findings demonstrated in object identification.

Though evidence for a repetition priming effect is ubiquitous, its mechanism remains open to question. Morton (1979) argued that the information processing of previous stimuli reduced the activation threshold of their representations in subsequent processing. Rouder et al. (2000) have suggested that priming effects are a bias in the post-perceptual decision stage. Finally, Roediger (2003) argued that the priming effect was an evolutionary adaptation to effective reprocessing of familiar stimuli. In contrast to these older theories, Marsolek and colleagues (Marsolek et al., 2006; Marsolek, 2008) proposed a new perspective of why priming effects are observed. They argue that the antipriming effect is caused by a measurable impaired processing of novel stimuli when the representations between new and old objects overlap, reflecting dynamic adjustments or maintenance relearning of overlapping representations. Results from the present study corroborated Marsolek and colleague's work with respect to object identification accuracy, and went further to show generalizability of the findings to Chinese character identification.

Differences in the response times between antiprimed characters and baseline in the experiment did not reach significance; these findings are consistent with results from the Marsolek et al. (2006) study. Although antiprimed characters become difficult to identify due to overlapping representations, it remains difficult to ascertain the impact of the visual antipriming effect on response times, because the analyses of response times in the experimental design only considered the trials of correctly identified objects. Future research may consider alternative experimental strategies to further investigate this possibility.

Importantly, the present experiment differed from previous work such as Spataro et al. (2017) in that there were no masks in the test phase. By removing masks in the test phase, we were able to minimize competing orthographic and phonological representations that would have otherwise been jointly activated before the participants completed the perceptual identification and lexical decision tasks. In this way participants could perceive the whole characters at a single time, allowing activation of all the visual form representations of primed and antiprimed characters prior to character identification.

With regard to lexical access, we would expect that when a given Chinese character (e.g., “枪”, meaning “gun”) was identified, another orthographically similar Chinese character (e.g., “棉”, meaning “cotton”) would be weakly activated because of the common feature (i.e., “木” of the left part of both the characters). In order to reduce the competition for final identification, the overlapping feature would be more sensitive to the identified characters (Marsolek, 2008). Further, due to the changes in connection strengths, a repetition-primed character would be re-identified more effectively and a character having a common orthographic feature would be re-identified less effectively. In this way, the antipriming effect could be related to the dynamic adjustments of overlapping representations. However, Huang et al. (2006) found that all words sharing the first constituent character are activated in the early stage of word recognition in reading two-character words. Given these findings, future research is needed to understand the way in which orthographic neighborhood contributes to the visual antipriming effect.

The magnitude of an antipriming effect is thought to relate to the degree of representation overlap or similarity between objects (Marsolek, 2008). In the present study, half of an antiprimed character was the same as a primed character, yielding a visual similarity of approximately 50%. This led to results showing that the magnitude of the antipriming effect (0.92–0.86 = 0.06) was similar to the repetition priming effect (0.97–0.92 = 0.05). These results contrast to earlier work in two ways. First, in previous work the magnitude of the antipriming effect was shown to be smaller than the priming effect (Marsolek et al., 2006). Second, the character identification accuracies in the present study were relatively higher than earlier studies. We suspect that these differences derive, in part, from the fact that the Chinese characters selected for use in the experiment were chosen to be easily identifiable and familiar to participants (frequency range: 1,507–3,373). Further, previous work did not test the objects' familiarities, or provide a report of visual similarity between antiprimed objects and primed objects (Marsolek et al., 2006). Given that research has demonstrated that the identification accuracy of antiprimed objects differs across high and low similarity conditions (Deason, 2008), we recommend that future research should consider manipulating the visual similarity of experimental stimuli as an experimental factor, as well as should incorporate Chinese characters unfamiliar to participants to further explore the antipriming effect in Chinese character identification.

## Conclusion

This study used Marsolek et al.'s (2006) novel repetition priming experiment paradigm to investigate the visual antipriming effect in Chinese character identification. Results demonstrated new support for the overlapping representation theory of knowledge, and indicated that Chinese character representations appear to be stored in an overlapping way. Specifically, strengthening character representations after usage resulted in the priming effect of that character, and strengthening character representations inclusive of common form features yielded an antipriming effect.

## Ethics statement

This study was carried out in accordance with the recommendations of Ethics Guidelines of Research Ethics Committee of Henan University with written informed consent from all subjects. All subjects gave written informed consent in accordance with the Declaration of Helsinki. The protocol was approved by the Research Ethics Committee of Henan University.

## Author contributions

The experiment was designed and performed by FZ. The manuscript was written by FZ and AF. The manuscript was also revised by XL.

### Conflict of interest statement

The authors declare that the research was conducted in the absence of any commercial or financial relationships that could be construed as a potential conflict of interest.

## Appexndix

### Experimental materials of Chinese characters

#### The first phase of the experiment: 50 Chinese character

横征途脸湿喜乱松套屋笔临顾架掉亮盖编塞促奏私呼怀盘恶播裁塔炉困麦盐龙货冷短错括罗欢夏倒网混重刘普吧哪

#### The second phase of the experiment: 100 Chinese character (baseline measurement)

零脚凯背贵顺康钟染弦班慢迫执延扬静剧触威秋缩弱航课药季岩裂努杆矛探希镇卷苗浓哈菌竟税煤灵鸡野耐透荷刻略泥遇愈彩圈盟哥旗献待磁补块胜沉穿宜督振胶衡蒸唯念迅筑校室雨唱善毫酒沙医模扩获察停粉夜轴益频州逐移聚

#### The third phase of the experiment: 50 Chinese character (priming stage)

枪侧堆退择铜握氢评燃践宪库培胞厚景绍雷饭伯赛座粗碳答遍幅赵晚挥终茶植顶粒守皇载渐孩突苦析银鲜硫村堂游

#### The fourth phase of the experiment: 100 Chinese character (testing stage)

Fifty Chinese characters are identical to the third stage. Another 50 Chinese characters are as follows:

棉倾雄追泽销措氯诗烧跳宗序倍肥愿星纯雪饲怕寨庭祖硬符偏福赶映控绝菜殖预粮夺息栽激核客若诉针洋础讨掌湖
